# Dose-response Associations of Physical Activity and Sitting Time With All-cause Mortality in Older Japanese Adults

**DOI:** 10.2188/jea.JE20220246

**Published:** 2024-01-05

**Authors:** Satoshi Seino, Takumi Abe, Yu Nofuji, Toshiki Hata, Shoji Shinkai, Akihiko Kitamura, Yoshinori Fujiwara

**Affiliations:** 1 Research Team for Social Participation and Community Health, Tokyo Metropolitan Institute of Gerontology, Tokyo, Japan; 2 Department of Food and Nutritional Science, Graduate School of Applied Bioscience, Tokyo University of Agriculture, Tokyo, Japan; 3 Department of Nutrition Sciences, Kagawa Nutrition University, Saitama, Japan; 4 Health Town Development Science Center, Osaka, Japan; 5 Integrated Research Initiative for Living Well with Dementia, Tokyo Metropolitan Institute of Gerontology, Tokyo, Japan

**Keywords:** physical activity, sitting time, mortality, dose-response, IPAQ-short, guidelines

## Abstract

**Purpose:**

Although examining the dose-response curves of physical activity (PA) and sitting time with health-related outcomes is an important research agenda, the results for older Japanese adults are extremely limited. We examined the dose-response associations of PA and sitting time with all-cause mortality among older Japanese.

**Methods:**

Initially, 8,069 non-disabled residents (4,073 men; 3,996 women) aged 65–84 years of Ota City, Japan, were analyzed. Moderate-to-vigorous PA (MVPA) and sitting time were evaluated using the International Physical Activity Questionnaire-Short Form. Multivariate-adjusted hazard ratios (HRs) and 95% confidence intervals (CIs) of MVPA and sitting time for all-cause mortality were calculated, and the dose-response curves were examined using restricted cubic splines (RCS).

**Results:**

During 4.1 years of follow-up, 458 participants (5.7%; 331 men and 127 women) died. Compared with the low MVPA (<600 metabolic equivalents [METs]·minutes/week) group, HR for mortality gradually reduced in moderate (600–3,000 METs·minutes/week) and high (>3,000 METs·minutes/week) MVPA groups (moderate: HR 0.66; 95% CI, 0.54–0.82; high: HR 0.58; 95% CI, 0.45–0.75; *P* < 0.001 for trend). RCS showed that the HR for mortality reduced linearly up to approximately 2,000 METs·minutes/week of MVPA, and maximal risk reduction was seen at approximately 3,000–4,500 METs·minutes/week of MVPA. No significant dose-response association of sitting time with mortality was observed.

**Conclusion:**

Higher MVPA levels reduced all-cause mortality risk, in a significant inverse non-linear dose-response manner. Sitting time was not significantly associated with all-cause mortality. It is important to disseminate the significance of even a slight increase in the MVPA for reducing mortality risk.

## INTRODUCTION

In the World Health Organization (WHO) 2020 guidelines on physical activity (PA) and sedentary behavior,^1^^,^^2^ extensive evidence of PA and public health recommendations were provided. As already known, PA significantly lowers all-cause mortality in both younger and older adults.^1^^,^^3^^,^^4^ The mechanisms are explained by the wide range of health benefits of PA, including reducing cardiovascular disease mortality, hypertension, type 2 diabetes, and site-specific cancers and improving cardiorespiratory and muscular fitness, reducing adiposity, improving sleep, reducing symptoms of anxiety and depression, and improving cognitive health.^1^^–^^4^ Particularly in older adults, PA also helps prevent falls and fall-related injuries,^1^ sarcopenia,^5^ and frailty.^6^

The 2020 WHO PA and Sedentary Behavior Guidelines Development Group has pointed out a lack of evidence on the precise dose-response curve between PA and/or sedentary behavior and health-related outcomes as one of the evidence gaps across population sub-groups.^7^ Although previous pooled^8^^,^^9^ or systematic reviews and meta-analyses^10^^,^^11^ examining these dose-response curve showed that higher levels of PA^8^^–^^11^ and less time spent in sedentary activities^10^ were significantly associated with reduced mortality in a non-linear dose-response manner, most of the data used in these studies^8^^–^^11^ were from Western countries. The number of such studies on the dose-response curve shape among Asian older adults, including Japan, is still limited.^12^^–^^14^ Moreover, a pooled analysis by the Asia Cohort Consortium,^15^ which examined the association of leisure-time PA categories with all-cause and cause-specific mortality, did not show a clear dose-response association. The population attributable fractions of physical inactivity to all-cause mortality in Japan (16.1%) was 1.7 times higher than the median of 122 countries (9.4%) worldwide.^16^ This suggests that there is a relatively stronger relationship between PA and life expectancy in Japanese than that in populations from other countries, and population-specific dose-response analyses may be required.

We examined the dose-response associations of PA and sitting time with all-cause mortality among community-dwelling older Japanese adults. Specifically, we aimed to clarify the shape of dose-response curves and the lowest thresholds of mortality risk of PA and sitting time, and the presence or absence of maximal safety thresholds of PA, which are important agenda to be examined.^7^

## METHODS

### Study participants

We used data from a community-wide intervention study on frailty prevention that was conducted in 2016, in Ota City, Tokyo, Japan.^17^^,^^18^ Details of the study participant selection process have already been published.^17^ Briefly, 15,500 residents aged 65–84 years were selected by sex and age group (65–74 and 75–84 years) random stratified sampling method from all 18 districts of the city.^17^ All participants were physically and cognitively independent—defined as the absence of long-term care insurance (LTCI) certification.^19^^,^^20^

**Figure 1.**
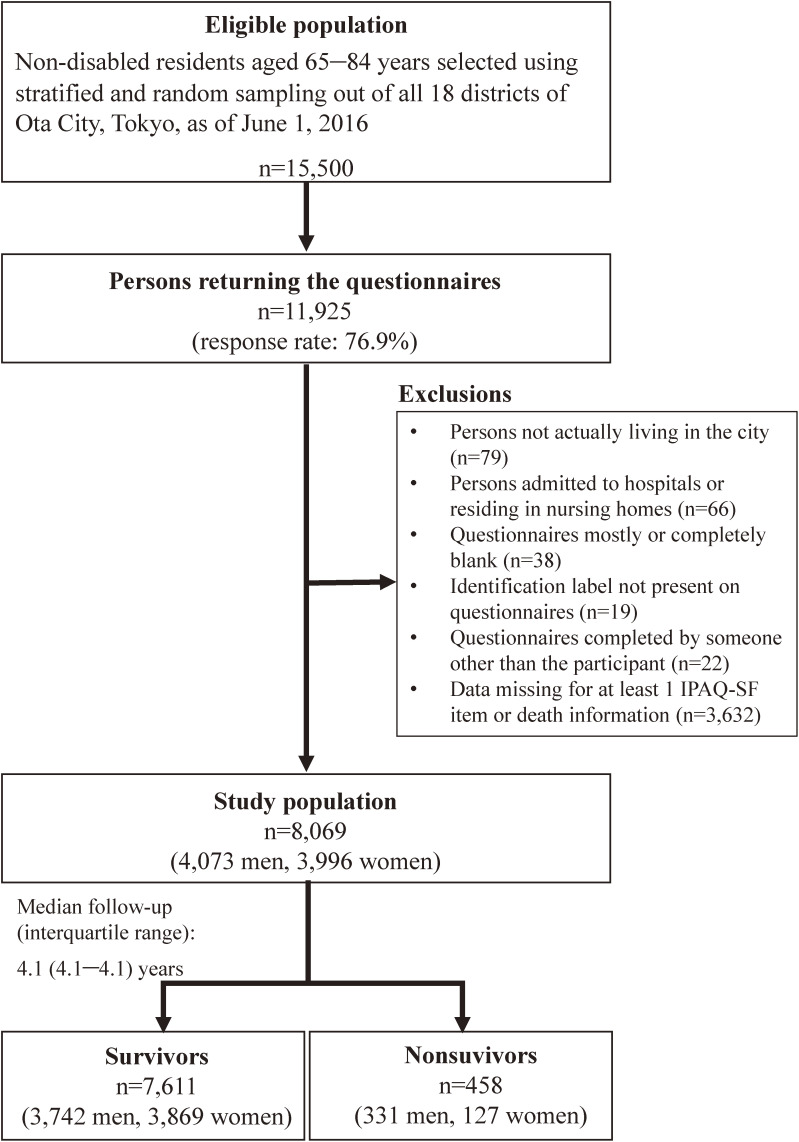
Flow diagram of the study participants. ^*^The 3,632 respondents we excluded from analysis include those who were excluded based on the Guidelines for Data Processing and Analysis of the IPAQ-SF.^23^ IPAQ-SF, International Physical Activity Questionnaire-Short Form.

Of the 15,500 self-administered questionnaires distributed in July 2016, 11,925 were returned (response rate: 76.9%). Finally, 8,069 questionnaires (from 4,073 men and 3,996 women) were included in this analysis (Figure 1).

We obtained ethical approval from the Ethical Committee of the Tokyo Metropolitan Institute of Gerontology (approved June 1, 2016). All participants were informed that participation in this study was voluntary and that returning the self-administered questionnaire via mail would indicate their consent to participate in this study.

### Measurements

#### PA and sitting time

Individuals’ levels of PA and sitting time were evaluated using the International Physical Activity Questionnaire-Short Form (IPAQ-SF; Japanese version).^21^^,^^22^ Participants were assessed based on their usual weekday sitting time, apart from the sleeping time, as well as their time spent performing vigorous-intensity PA (VPA), moderate-intensity PA (MPA), and walking time during a typical week. All missing values and outliers were treated according to the Guidelines for Data Processing and Analysis of the IPAQ-SF.^23^ Moreover, in the analysis of sitting time, answers with 0 minutes were regarded as missing values. All instances where the sitting time was >1,080 minutes (18 hours) per day were replaced with 1,080 minutes. We defined total moderate-to-vigorous PA (MVPA) as 7 days × (8.0 metabolic equivalents [METs] × vigorous PA minutes/day + 4.0 METs × moderate PA minutes/day + 3.3 METs × walking time minutes/day).^23^ The total MVPA was categorized as low (<600 METs·minutes/week; ie, equivalent to <150 minutes/week of moderate intensity PA), moderate (600–3,000 METs·minutes/week; ie, equivalent to 150–750 minutes/week of moderate intensity PA), and high (>3,000 METs·minutes/week; ie, equivalent to >750 minutes/week of moderate intensity PA), in accordance with a previous study.^9^ The sitting times were categorized as <180, 180–299, 300–479, and ≥480 minutes/day, based on a previous study^24^ as well as on the median (300 minutes/day) in this study.

#### All-cause mortality

We ascertained the occurrence of all deaths on July 31, 2020, in Ota City, by checking local registries that had linked records with the Japanese National Vital Statistics System. Although new LTCI applications as of July 31, 2020 were affected by the novel coronavirus disease (COVID-19) pandemic,^25^ the impact of the COVID-19 pandemic on mortality was negligible at this point. Therefore, we set the endpoint of this study as July 31, 2020.

#### Covariates

The covariates included age, sex, district, living situation (living with others or alone), marital status (married, widowed, divorced, or never married), education (junior high school, high school, or junior college/vocational college/college/graduate school graduate), equivalent income (<2.0, 2.0–3.99, ≥4.0 million yen, or unknown), body mass index (BMI; <18.5, 18.5–24.9, or ≥25 kg/m^2^), hypertension, dyslipidemia, heart disease, stroke, diabetes mellitus, cancer, alcohol drinking and tobacco smoking statuses (current, never, or former), lower-back pain, and knee pain (presence or absence). Equivalent income was calculated by dividing the household income by the square root of the number of household members.^26^ BMI was defined as self-rated body weight (kg) divided by self-rated height squared (m^2^). To reduce selection bias, covariates with missing data were assigned to the “missing” category and included in the analysis.

### Statistical analyses

Data were analyzed using Stata 17.0 (StataCorp, College Station, TX, USA). An *α* of 0.05 indicated statistical significance. Descriptive statistics was calculated to characterize the study participants. The unpaired *t* test, Mann–Whitney U test, or chi-square test were used to compare the survivors and non-survivors’ baseline characteristics.

For primary analysis, we performed Cox proportional hazard models with all-cause mortality as the dependent variable and MVPA or sitting time categories as independent variables. The follow-up person-years were calculated for each participant from July 1, 2016, until the date of death, migration from Ota City, or the end of the follow-up period (July 31, 2020), whichever occurred first. We constructed three analyses models. Model 1 was adjusted for age and sex. Model 2 additionally adjusted for district, living situation, marital status, educational attainment, equivalent income, BMI, hypertension, dyslipidemia, heart disease, stroke, diabetes mellitus, cancer, alcohol consumption and tobacco smoking statuses, lower-back pain, and knee pain. Model 3 was further adjusted for either MVPA or sitting time according to the independent variable. To examine whether the results varied by sex, the statistical interaction was tested between the MVPA or sitting time categories and by sex.

Furthermore, we examined the dose-response curves of MVPA and sitting time with mortality risk in model 3 using restricted cubic splines following the same procedures as in previous studies.^27^^,^^28^ We used the Akaike information criterion (AIC) to select the restricted cubic spline with 3, 4, or 5 knots and adopted the model with the lowest AIC.^29^ We set the MVPA of 0 METs·minutes/week or median sitting time (300 minutes/day) as the reference value for each model.

We performed two sensitivity analyses by using the same statistical approaches that were conducted as in the main analysis. First, to completely exclude any possible impacts of the COVID-19 pandemic on PA and mortality during the follow-up period, we set January 31, 2020 (before the COVID-19 pandemic reached Japan) as the endpoint (sensitivity analysis 1). Second, to reduce possible reverse causation, we excluded deaths during the first 2 years of follow-up (sensitivity analysis 2) because a previous study has pointed out that at least a 2-year lag period is needed to minimize the impact of reverse causation on the association of PA with mortality.^30^ Moreover, to clarify if the dose-response associations of MVPA and sitting time with mortality are differ between age groups, we performed stratified analyses for participants by two age groups, 65–74 years and 75–84 years, using the same statistical approaches.

## RESULTS

Of the 8,069 people for whom the status of mortality could be confirmed (follow-up rate of 99.98%), 458 (5.7%; 331 men and 127 women) participants died, with rates of 14.4 per 1,000 person-years. Of these, 168 (36.7%; 129 [39.0%] men and 39 [30.7%] women) died during the first 2 years, and 407 (88.9%; 296 [89.4%] men and 111 [87.4%] women) died by January 31, 2020 (before the COVID-19 pandemic in Japan).

Table 1 indicates the baseline characteristics of the study population, by survival status. The medians of the VPA, MPA, walking time, MVPA and sitting time were 0 (interquartile range [IQR], 0–80) minutes/week, 0 (IQR, 0–120) minutes/week, 300 (IQR, 120–560) minutes/week, 1,485 (IQR, 594–3,324) METs·minutes/week and 300 (IQR, 180–480) minutes/day, respectively. Compared to survivors, non-survivors were significantly older, more likely to be men, married, and current smokers. Moreover, they had lower MVPA, educational attainment, equivalent income, BMI, and lower prevalence of dyslipidemia and current drinking. They also had higher sitting time, higher prevalence of hypertension, heart disease, stroke, diabetes mellitus, cancer, and lower-back pain.

**Table 1.** Baseline characteristics of the study population, by survival status

	All		Survivors		Nonsurvivors		*P*
	(*n* = 8,069)		(*n* = 7,611)		(*n* = 458)		
**VPA, min/week, median (interquartile range)**	0	(0–80)	0	(0–80)	0	(0–60)	0.20
**MPA, min/week, median (interquartile range)**	0	(0–120)	0	(0–120)	0	(0–0)	<0.001
**Walking time, min/week, median (interquartile range)**	300	(120–560)	300	(120–600)	180	(30–420)	<0.001
**MVPA, METs·min/week, median (interquartile range)**	1,485	(594–3,324)	1,485	(594–3,360)	990	(198–2,616)	<0.001
Low (<600 METs·min/week), *n* (%)	2,103	(26.1)	1,918	(25.2)	185	(40.4)	<0.001
Moderate (600–3,000 METs·min/week), *n* (%)	3,778	(46.8)	3,595	(47.2)	183	(40.0)	
High (>3,000 METs·min/week), *n* (%)	2,188	(27.1)	2,098	(27.6)	90	(19.7)	
**Sitting time, min/day, median (interquartile range)**	300	(180–480)	300	(180–480)	360	(210–540)	0.003
<180, *n* (%)	1,408	(17.5)	1,334	(17.5)	74	(16.2)	0.013
180–299, *n* (%)	1,927	(23.9)	1,842	(24.2)	85	(18.6)	
300–479, *n* (%)	2,214	(27.4)	2,082	(27.4)	132	(28.8)	
≥480, *n* (%)	2,520	(31.2)	2,353	(30.9)	167	(36.5)	
**Age, years, mean (SD)**	73.8	(5.5)	73.7	(5.5)	76.1	(5.3)	<0.001
**Sex, men, *n* (%)**	4,073	(50.5)	3,742	(49.2)	331	(72.3)	<0.001
**Living alone, *n* (%)**	1,611	(20.0)	1,512	(19.9)	99	(21.6)	0.61
**Marital status, *n* (%)**							0.033
Married	5,461	(67.7)	5,139	(67.5)	322	(70.3)	
Widowed or divorced	1,888	(23.4)	1,802	(23.7)	86	(18.8)	
Never married	616	(7.6)	576	(7.6)	40	(8.7)	
**Education, *n* (%)**							0.001
Junior high school graduation	1,750	(21.7)	1,620	(21.3)	130	(28.4)	
High school graduation	3,024	(37.5)	2,876	(37.8)	148	(32.3)	
Junior college/vocational college/ ​ college/graduate school graduation	3,068	(38.0)	2,907	(38.2)	161	(35.2)	
Other/missing	227	(2.8)	208	(2.7)	19	(4.2)	
**Equivalent income, *n* (%)**							0.047
<2.0 million yen	1,336	(16.6)	1,258	(16.5)	78	(17.0)	
2.0–3.99 million yen	2,888	(35.8)	2,699	(35.5)	189	(41.3)	
≥4.0 million yen	2,412	(29.9)	2,289	(30.1)	123	(26.9)	
Unknown/missing	1,433	(17.8)	1,365	(17.9)	68	(14.9)	
**BMI, kg/m^2^, mean (SD)**	22.7	(3.1)	22.7	(3.1)	22.4	(3.4)	0.020
<18.5, *n* (%)	650	(8.1)	590	(7.8)	60	(13.1)	<0.001
18.5–24.9, *n* (%)	5,672	(70.3)	5,372	(70.6)	300	(65.5)	
≥25, *n* (%)	1,696	(21.0)	1,605	(21.1)	91	(19.9)	
**Hypertension, *n* (%)**	4,266	(52.9)	4,007	(52.7)	259	(56.6)	<0.001
**Dyslipidemia, *n* (%)**	3,327	(41.2)	3,177	(41.7)	150	(32.8)	<0.001
**Heart disease, *n* (%)**	1,772	(22.0)	1,621	(21.3)	151	(33.0)	<0.001
**Stroke, *n* (%)**	593	(7.4)	545	(7.2)	48	(10.5)	0.001
**Diabetes mellitus, *n* (%)**	1,434	(17.8)	1,313	(17.3)	121	(26.4)	<0.001
**Cancer, *n* (%)**	1,304	(16.2)	1,152	(15.1)	152	(33.2)	<0.001
**Alcohol drinking status, current, *n* (%)**	4,544	(56.3)	4,312	(56.7)	232	(50.7)	<0.001
**Smoking status, current, *n* (%)**	1,030	(12.8)	945	(12.4)	85	(18.6)	<0.001
**Lower-back pain, *n* (%)**	3,054	(37.9)	2,854	(37.5)	200	(43.7)	0.008
**Knee pain, *n* (%)**	2,437	(30.2)	2,306	(30.3)	131	(28.6)	0.31

BMI, body mass index; METs, metabolic equivalents; MPA, moderate-intensity physical activity; MVPA, moderate-to-vigorous physical activity; SD, standard deviation; VPA, vigorous-intensity physical activity.

Table 2 exhibits multivariate-adjusted hazards ratio (HRs) and 95% confidence intervals (CIs) of MVPA and sitting time for all-cause mortality. Compared with the low MVPA group, the moderate (HR 0.66; 95% CI, 0.54–0.82) and high (HR 0.58; 95% CI, 0.45–0.75) MVPA groups had significantly and gradually reduced mortality, even in the fully adjusted model 3 (*P* < 0.001 for trend). No significant relationship between sitting time and mortality was observed in either model. These results of MVPA and sitting time did not vary by sex (*P* for interactions >0.309 for all models). The results of the two sensitivity analyses that excluded the period of COVID-19 pandemic spread (sensitivity analysis 1) (eTable 1) and deaths during the first 2 years of follow-up (sensitivity analysis 2) (eTable 2) and the stratified analyses for participants aged 65–74 years (eTable 3) and 75–84 years (eTable 4) were not substantially different from those of the primary analyses.

**Table 2.** Multivariate-adjusted HRs and 95% CIs of MVPA and sitting time for all-cause mortality (*n* = 8,069)

Variables	Number of events per participants	Incidence rate per 1,000 person-years	Model 1			Model 2			Model 3		
		
			HR	(95% CI)	*P*	HR	(95% CI)	*P*	HR	(95% CI)	*P*
**MVPA**											
Low ​ (<600 METs·min/week)	185/2,103	22.7	1.00	(Ref.)		1.00	(Ref.)		1.00	(Ref.)	
Moderate ​ (600–3,000 METs·min/week)	183/3,778	12.2	0.59	(0.48–0.72)	<0.001	0.66	(0.53–0.81)	<0.001	0.66	(0.54–0.82)	<0.001
High ​ (>3,000 METs·min/week)	90/2,188	10.4	0.51	(0.40–0.66)	<0.001	0.57	(0.44–0.73)	<0.001	0.58	(0.45–0.75)	<0.001
	458/8,069	14.4	Trend		<0.001	Trend		<0.001	Trend		<0.001
**Sitting time**											
<180 min/day	74/1,408	13.3	1.00	(Ref.)		1.00	(Ref.)		1.00	(Ref.)	
180–299 min/day	85/1,927	11.1	0.83	(0.61–1.13)	0.230	0.78	(0.57–1.07)	0.118	0.78	(0.57–1.06)	0.112
300–479 min/day	132/2,214	15.2	1.09	(0.82–1.46)	0.533	1.06	(0.79–1.41)	0.703	1.03	(0.77–1.37)	0.864
≥480 min/day	167/2,520	16.9	1.14	(0.87–1.50)	0.344	1.06	(0.80–1.40)	0.700	1.00	(0.76–1.32)	0.988
	458/8,069	14.4	Trend		0.072	Trend		0.180	Trend		0.389

CI, confidence interval; HR, hazard ratio; METs, metabolic equivalents; MVPA, moderate-to-vigorous physical activity.

Model 1: Adjusted for baseline age and sex.

Model 2: Adjusted for variables in model 1 plus district, living situation, marital status, education, equivalent income, body mass index, hypertension, dyslipidemia, heart disease, stroke, diabetes mellitus, cancer, alcohol drinking status, smoking status, lower-back pain, and knee pain.

Model 3: For MVPA, adjusted for variables in model 2 plus sitting time. For sitting time, adjusted for variables in model 2 plus MVPA.

**Figure 2.**
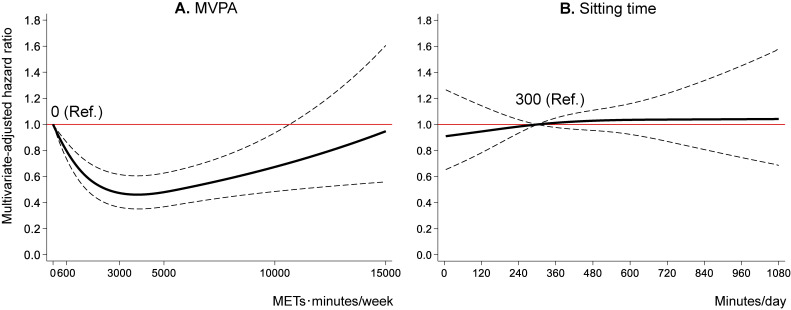
Dose-response relationship of MVPA and sitting time with all-cause mortality. Figure 2 shows the relationships of (**A**) MVPA and (**B**) sitting time with all-cause mortality modeled by restricted cubic splines. MVPA and sitting time were mutually adjusted as well as the baseline age, sex, district, living situation, marital status, education, equivalent income, body mass index, hypertension, dyslipidemia, heart disease, stroke, diabetes mellitus, cancer, alcohol drinking status, smoking status, lower-back pain, and knee pain. The reference values for MVPA and sitting time were 0 METs·minutes/week and 300 minutes/day, respectively. The solid lines indicate the hazard ratios for all-cause mortality. The dashed lines indicate the 95% confidence intervals. METs, metabolic equivalents; MVPA, moderate-to-vigorous physical activity.

Figure 2 shows the results of the primary analyses of the dose-response curves of MVPA (Figure 2A) and sitting time (Figure 2B) with all-cause mortality in the fully adjusted model 3. Compared with no MVPA (the reference), the HR for mortality reduced linearly up to approximately 2,000 METs·minutes/week of MVPA, and the lowest HR (HR 0.46; 95% CI, 0.35–0.61) were reached at around 3,000–4,500 METs·minutes/week (Figure 2A). However, the lower HRs became modest above that MVPA (eg, the HR at 10,000 METs·minutes/week, 0.67; 95% CI, 0.48–0.93). The results showed increased uncertainty, as reflected by the wide 95% CIs, at higher (approximately 10,000 METs·minutes/week and above) MVPA. No significant dose-response relationship was seen in the sitting time (Figure 2B), and the result was consistent even when analyzed by MVPA level (eFigure 1). The shapes of the dose-response curves of MVPA (eFigure 2) and sitting time (eFigure 3) in the sensitivity and stratified analyses were not substantially different from those of the primary analyses.

## DISCUSSION

A significant inverse non-linear dose-response relationship was identified between MVPA, assessed by IPAQ-short, and all-cause mortality. All-cause mortality risk reduced linearly up to approximately 2,000 METs·minutes/week of MVPA, and maximal risk reductions were consistently seen at approximately 3,000–4,500 METs·minutes/week of MVPA in the primary, sensitivity, and stratified analyses. Although PA levels above that MVPA did not further reduce mortality risk, we did not identify a significantly higher mortality risk at any higher level of MVPA in either analysis. Finally, no significant dose-response relationship between sitting time and mortality risk was observed.

Our primary analysis results showed a somewhat inverse J-shaped association of MVPA with all-cause mortality. However, reverse causation bias and regression dilution bias due to measurement error should be noted in cohort studies with relatively short follow-up periods and with a self-reported measure of PA at baseline alone,^30^^,^^31^ as in this study. People with pre-existing or undiagnosed diseases that increase the risk of mortality may become inactive, which would lead to an overestimation of the true inverse association especially between lower MVPA ranges and mortality.^31^ Lee et al^30^ examined how the use of single measure versus repeated measures of PA and how different time lags (period from PA assessment to death) affected the association of PA with mortality. A single measure of PA at baseline was more likely to show an inverse J-shaped or somewhat U-shaped dose-response associations, while the cumulative average by repeated measures of PA was consistently inversely associated with mortality across all PA ranges.^30^ They suggested that excluding deaths in the first year may be inadequate to reduce reverse causation, and that at least a 2-year lag period is required to minimize reverse causation bias.^30^^,^^31^ In our sensitivity analysis 2, the lower HRs tended to be maintained even at higher MVPAs compared to the primary analysis. Considering these, the somewhat increasing trends after 4,500 METs·minutes/week of MVPA seen in the primary analysis of this study may not be due to a true biological effect, but could be due to measurement error due to a single assessment at baseline and/or reverse causation biases.

A concern of recent studies is the determination of the maximal safety thresholds of MVPA.^7^ A detailed pooled analysis,^8^ and a systematic review and meta-analysis^11^ consistently show that compared to the recommended level of PA, mortality risk is lower at PA levels well above the recommended level, and no threshold will increase the mortality risk beyond the recommended level. Moreover, in the PURE study, which included PA data from 17 countries assessed using IPAQ-long form,^9^ the HR for mortality significantly reduced up to approximately 3,000 METs·minutes/week and the significant benefits remained even between 5,000–15,000 METs·minutes/week. Our results of the MVPA with the lowest mortality (3,000–4,500 METs·minutes/week) and PA guideline (ie, MVPA of ≥150 minutes/week in bouts of ≥10 minutes)^32^ sufficiency rate of 78.6% were generally similar to the results of the PURE study.^9^ Furthermore, the results that did not significantly increase the mortality risk even at MVPA above the recommended levels were also consistent with these previous studies.^8^^,^^9^^,^^11^ Our results do not seem to be evidence that the upper limit of MVPA should be set among older adults.

The 3,000–4,500 METs·minutes/week (ie, equivalent to 50–75 METs·hours/week), which achieved the lowest HR for mortality in our study, corresponds to more than twice the recommended value (ie, 23 METs·hours/week of PA with an intensity of ≥3 METs) for Japanese adults aged 18–64 years,^33^ or approximately 5–7.5 times the recommended value (ie, 10 MET·hours/week of PA regardless of an intensity) for Japanese adults aged 65 years or older.^33^ Because self-reported PA instruments, such as IPAQ, tend to overestimate PA as compared to more valid objective devices,^22^ the true value of the lowest mortality risk threshold may be lower. However, a recent study using tri-axial accelerometer^34^ reported that 71.0% of older Japanese adults had already met the current Japanese PA guideline for older adults^33^ with only their MVPA. Further considering the additional health benefits^1^ and no harmful effects^8^^,^^9^^,^^11^ of higher level of PA, we believe that at least higher level of PA standards than current Japanese PA guideline^33^ should be recommended for older Japanese adults.

Many previous studies^10^^,^^24^^,^^35^ have reported a positive association of sitting time with mortality risk, while several studies have shown the absence of a significant association,^36^^–^^38^ including our results. This tendency seems to be more pronounced in more active^39^ or less frail^40^ populations. However, in our study, sitting time was not consistently associated with all-cause mortality, regardless of the MVPA level (eFigure 1). In our population, the all-cause mortality risk may have been suppressed depending on the type of sedentary behavior (mentally-active or passive).^41^ Future research needs to take such differences in types of sedentary behaviors into consideration.

Our findings might also be attributable to cognitive biases due to sitting time evaluation using IPAQ-short. Self-reported sedentary time is often underestimated.^42^ The median sitting time (300 minutes/day) as assessed using IPAQ-short in this study was substantially lower than that assessed using tri-axial accelerometer (478.0 minutes/day in men and 417.9 minutes/day in women).^34^ Moreover, the IPAQ sitting time question may have been difficult to answer for some older adults.^43^ It may have been desirable to ask the categories of sitting time^24^ or TV viewing time^35^^,^^44^ to reasonably assess the association with mortality.

Other limitations warrant mention. First, selection bias is a concern. The exclusion of the 3,632 (31%) respondents with missing IPAQ-SF data may have skewed the analysis to a health-conscious population. Second, our study was limited to older adults living in a metropolitan area, which may limit the generalization. Third, the 4-year follow-up period was relatively short. However, follow-up after the endpoint set in this study was concerning because the COVID-19 epidemic would have a greater impact on reduced PA,^45^ and it may also affect subsequent mortality. With this in mind, we have set this as the minimum required follow-up period. Although we performed sensitivity analyses that excluded deaths during the first 2 years as well as the exclusion of the COVID-19 period, possible reverse causation cannot be completely excluded in our study. Fourth, mortality rate was low especially in women. Ideally, analyses excluding baseline fatal diseases were desirable because the multivariate-adjusted model in which prevalent diseases were not excluded had a stronger inverse association of PA with health-related outcomes than when they were excluded.^31^ However, due to the limited number of events, we were unable to perform such analyses. These should be analyzed in future research. Finally, it should be mentioned that this study used data from a community-wide intervention that included PA promotion.^18^ Although the intervention did not affect population-level frailty at 2 years^18^ and all-cause mortality during the current follow-up period (data not shown), the walking time in the intervention subgroup improved at the population-level.^18^ The MVPA was evaluated at baseline only, and we were unable to consider changes during the follow-up period. If MVPA had improved further during the subsequent follow-up period after 2 years, the associations of MVPA with mortality risk shown in this study may have been underestimated.

Despite these limitations, our study is strengthened by the relatively large scale of randomly recruited participants and the high response and follow-up rates. Our results can contribute to the construction of future PA guidelines for older adults.

### Conclusion

Higher levels of MVPA reduced the all-cause mortality risk, especially at well above recommended levels, in a significant inverse non-linear dose-response manner. There was no significant dose-response association of sitting time with mortality risk in this study. Although it may not be easy to reach the MVPA level associated with the lowest mortality (>3,000 METs·minutes/week) immediately, even a small increase in MVPA (up to approximately 2,000 METs·minutes/week) reduced the all-cause mortality risk. It is important to disseminate the significance of even a slight increase in the MVPA.

## SUPPLEMENTARY MATERIAL

eFigure 1. Dose-response relationship of sitting time with all-cause mortality across the MVPA categories: low (**A**), moderate (**B**), and high (**C**) MVPA were <600 METs·minute/week, 600–3,000 METs·minute/week, and >3,000 METs·minute/week, respectively. All models were adjusted for baseline age, sex, district, living situation, marital status, education, equivalent income, body mass index, hypertension, dyslipidemia, heart disease, stroke, diabetes mellitus, cancer, alcohol drinking status, smoking status, lower-back pain, and knee pain. The reference values for sitting time were 300 minutes/day. The solid lines indicate the hazard ratios for all-cause mortality. The dashed lines indicate the 95% confidence intervals. METs, metabolic equivalents; MVPA, moderate-to-vigorous physical activity.

eFigure 2. Sensitivity and stratified analyses of dose-response relationships of MVPA with all-cause mortality. eFigure 2 shows the results of the sensitivity analyses after (**A**) excluding the period of the COVID-19 pandemic (n = 8,069) and (**B**) excluding deaths during the first 2 years of follow-up (n = 7,901), as well as the stratified analyses by two age groups of (**C**) 65–74 years (n = 4,081) and (**D**) 75–84 years (n = 3,988). All models were adjusted for the baseline age, sex, district, living situation, marital status, education, equivalent income, body mass index, hypertension, dyslipidemia, heart disease, stroke, diabetes mellitus, cancer, alcohol drinking status, smoking status, lower-back pain, knee pain, and sitting time. The reference value for each model was 0 METs·minutes/week. The solid lines indicate the hazard ratios for mortality. The dashed lines indicate the 95% confidence intervals. COVID-19, novel coronavirus disease 2019; METs, metabolic equivalents; MVPA, moderate-to-vigorous physical activity.

eFigure 3. Sensitivity and stratified analyses of dose-response relationships of sitting time with all-cause mortality. eFigure 3 shows the results of the sensitivity analyses after (**A**) excluding the period of the COVID-19 pandemic (n = 8,069) and (**B**) excluding deaths during the first 2 years of follow-up (n = 7,901), as well as the stratified analyses of two age groups of (**C**) 65–74 years (n = 4,081) and (**D**) 75–84 years (n = 3,988). All models were adjusted for the baseline age, sex, district, living situation, marital status, education, equivalent income, body mass index, hypertension, dyslipidemia, heart disease, stroke, diabetes mellitus, cancer, alcohol drinking status, smoking status, lower-back pain, knee pain, and MVPA. The reference value for each model was 300 minutes/day. The solid lines indicate the hazard ratios for mortality. The dashed lines indicate the 95% confidence intervals. COVID-19, novel coronavirus disease 2019; MVPA, moderate-to-vigorous physical activity.

eTable 1. Multivariate-adjusted HRs and 95% CIs of MVPA and sitting time for all-cause mortality (the analysis endpoint was on January 31, 2020, n = 8,069)

eTable 2. Multivariate-adjusted HRs and 95% CIs of MVPA and sitting time for all-cause mortality, excluding deaths that occurred during the first 2 years of follow-up (n = 7,901)

eTable 3. Multivariate-adjusted HRs and 95% CIs of MVPA and sitting time for all-cause mortality (results for 65–74 years, n = 4,081)

eTable 4. Multivariate-adjusted HRs and 95% CIs of MVPA and sitting time for all-cause mortality (results for 75–84 years, n = 3,988)
