# Supplementary material for: Dose-response Associations of Physical Activity and Sitting Time With All-cause Mortality in Older Japanese Adults
Source: J Epidemiol. 2024 Jan 5;34(1):23–30. doi: 10.2188/jea.JE20220246 (PMC10701252; doi:10.2188/jea.JE20220246)
Supplement: Supplementary file 1 [file je-34-023-s001.pdf]

eTable 1. Multivariate-adjusted HRs and 95% CIs of MVPA and sitting time for all-cause mortality (the analysis endpoint was on January 31, 2020, n=8,069)

Variables	Number of events per participants	Incidence rate per 1,000 person-years	Model 1			Model 2			Model 3		
			HR	(95% CI)	*P*	HR	(95% CI)	*P*	HR	(95% CI)	*P*
**MVPA**											
Low (<600 METs･min/week)	165/2,103	22.9	1.00	(Ref.)		1.00	(Ref.)		1.00	(Ref.)	
Moderate (600–3,000 METs･min/week)	161/3,778	12.2	0.58	(0.47–0.72)	<0.001	0.65	(0.52–0.81)	<0.001	0.66	(0.53–0.82)	<0.001
High (>3,000 METs･min/week)	81/2,188	10.6	0.52	(0.40–0.67)	<0.001	0.57	(0.43–0.74)	<0.001	0.58	(0.44–0.76)	<0.001
	407/8,069	14.5		Trend	<0.001		Trend	<0.001		Trend	<0.001
**Sitting time**											
<180 min/day	67/1,408	13.7	1.00	(Ref.)		1.00	(Ref.)		1.00	(Ref.)	
180–299 min/day	75/1,927	11.1	0.81	(0.58–1.12)	0.204	0.76	(0.54–1.06)	0.104	0.76	(0.54–1.05)	0.098
300–479 min/day	117/2,214	15.2	1.08	(0.80–1.45)	0.631	1.03	(0.76–1.40)	0.851	1.00	(0.74–1.35)	0.997
≥480 min/day	148/2,520	16.9	1.12	(0.84–1.50)	0.431	1.04	(0.77–1.39)	0.812	0.98	(0.73–1.31)	0.878
	407/8,069	14.5		Trend	0.104		Trend	0.242		Trend	0.481

CI, confidence interval; HR, hazard ratio; METs, metabolic equivalents; MVPA, moderate-to-vigorous physical activity.

Model 1: Adjusted for baseline age and sex.

Model 2: Adjusted for variables in model 1 plus district, living situation, marital status, education, equivalent income, body mass index, hypertension, dyslipidemia, heart disease, stroke, diabetes mellitus, cancer, alcohol drinking status, smoking status, lower-back pain, and knee pain.

eTable 2. Multivariate-adjusted HRs and 95% CIs of MVPA and sitting time for all-cause mortality, excluding deaths that occurred during the first 2 years of follow-up (n=7,901)

Variables	Number of events per participants	Incidence rate per 1,000 person-years	Model 1			Model 2			Model 3		
			HR	(95% CI)	*P*	HR	(95% CI)	*P*	HR	(95% CI)	*P*
**MVPA**											
Low (<600 METs･min/week)	112/2,030	13.9	1.00	(Ref.)		1.00	(Ref.)		1.00	(Ref.)	
Moderate (600–3,000 METs･min/week)	124/3,719	8.3	0.65	(0.51–0.84)	<0.001	0.73	(0.56–0.94)	0.016	0.73	(0.56–0.95)	0.018
High (>3,000 METs･min/week)	54/2,152	6.3	0.51	(0.37–0.70)	<0.001	0.55	(0.40–0.77)	<0.001	0.56	(0.40–0.78)	0.001
	290/7,901	9.2		Trend	<0.001		Trend	<0.001		Trend	<0.001
**Sitting time**											
<180 min/day	48/1,382	8.7	1.00	(Ref.)		1.00	(Ref.)		1.00	(Ref.)	
180–299 min/day	54/1,896	7.1	0.80	(0.54–1.18)	0.257	0.75	(0.50–1.11)	0.145	0.74	(0.50–1.09)	0.129
300–479 min/day	87/2,169	10.0	1.09	(0.77–1.56)	0.621	1.07	(0.75–1.53)	0.720	1.03	(0.72–1.47)	0.871
≥480 min/day	101/2,454	10.3	1.04	(0.74–1.47)	0.808	0.99	(0.70–1.40)	0.935	0.93	(0.65–1.32)	0.676
	290/7,901	9.2		Trend	0.348		Trend	0.457		Trend	0.718

CI, confidence interval; HR, hazard ratio; METs, metabolic equivalents; MVPA, moderate-to-vigorous physical activity.

Model 1: Adjusted for baseline age and sex.

Model 2: Adjusted for variables in model 1 plus district, living situation, marital status, education, equivalent income, body mass index, hypertension, dyslipidemia, heart disease, stroke, diabetes mellitus, cancer, alcohol drinking status, smoking status, lower-back pain, and knee pain.

Model 3: For MVPA, adjusted for variables in model 2 plus sitting time. For sitting time, adjusted for variables in model 2 plus MVPA.

eTable 3. Multivariate-adjusted HRs and 95% CIs of MVPA and sitting time for all-cause mortality (results for 65–74 years, n=4,081)

Variables	Number of events per participants	Incidence rate per 1,000 person-years	Model 1			Model 2			Model 3		
			HR	(95% CI)	*P*	HR	(95% CI)	*P*	HR	(95% CI)	*P*
**MVPA**											
Low (<600 METs･min/week)	53/966	14.1	1.00	(Ref.)		1.00	(Ref.)		1.00	(Ref.)	
Moderate (600–3,000 METs･min/week)	69/1,946	8.9	0.67	(0.47–0.96)	0.028	0.83	(0.57–1.21)	0.338	0.84	(0.57–1.23)	0.363
High (>3,000 METs･min/week)	31/1,169	6.6	0.50	(0.32–0.78)	0.002	0.59	(0.38–0.94)	0.026	0.60	(0.38–0.95)	0.030
	153/4,081	9.5		Trend	0.002		Trend	0.026		Trend	0.028
**Sitting time**											
<180 min/day	26/790	8.3	1.00	(Ref.)		1.00	(Ref.)		1.00	(Ref.)	
180–299 min/day	37/1,057	8.8	1.08	(0.65–1.78)	0.777	0.96	(0.58–1.59)	0.866	0.96	(0.57–1.59)	0.866
300–479 min/day	36/1,081	8.4	1.04	(0.63–1.73)	0.781	0.88	(0.52–1.48)	0.638	0.85	(0.51–1.44)	0.551
≥480 min/day	54/1,153	11.8	1.38	(0.86–2.20)	0.177	1.09	(0.67–1.76)	0.727	1.01	(0.62–1.65)	0.959
	153/4,081	9.5		Trend	0.162		Trend	0.691			0.969

CI, confidence interval; HR, hazard ratio; METs, metabolic equivalents; MVPA, moderate-to-vigorous physical activity.

Model 1: Adjusted for baseline age and sex.

Model 2: Adjusted for variables in model 1 plus district, living situation, marital status, education, equivalent income, body mass index, hypertension, dyslipidemia, heart disease, stroke, diabetes mellitus, alcohol drinking status, smoking status, lower-back pain, and knee pain.

Model 3: For MVPA, adjusted for variables in model 2 plus sitting time. For sitting time, adjusted for variables in model 2 plus MVPA.

eTable 4. Multivariate-adjusted HRs and 95% CIs of MVPA and sitting time for all-cause mortality (results for 75–84 years, n=3,988)

Variables	Number of events per participants	Incidence rate per 1,000 person-years	Model 1			Model 2			Model 3		
			HR	(95% CI)	*P*	HR	(95% CI)	*P*	HR	(95% CI)	*P*
**MVPA**											
Low (<600 METs･min/week)	132/1,137	30.1	1.00	(Ref.)		1.00	(Ref.)		1.00	(Ref.)	
Moderate (600–3,000 METs･min/week)	114/1,832	15.7	0.55	(0.43–0.71)	<0.001	0.59	(0.45–0.76)	<0.001	0.59	(0.46–0.76)	<0.001
High (>3,000 METs･min/week)	59/1,019	14.8	0.53	(0.39–0.72)	<0.001	0.58	(0.42–0.80)	0.001	0.59	(0.43–0.80)	0.001
	305/3,988	19.5		Trend	<0.001		Trend	<0.001		Trend	<0.001
**Sitting time**											
<180 min/day	48/618	20.0	1.00	(Ref.)		1.00	(Ref.)		1.00	(Ref.)	
180–299 min/day	48/870	13.9	0.70	(0.47–1.04)	0.076	0.64	(0.43–0.96)	0.032	0.63	(0.42–0.95)	0.026
300–479 min/day	96/1,133	21.7	1.10	(0.78–1.55)	0.606	1.08	(0.76–1.53)	0.686	1.03	(0.73–1.47)	0.853
≥480 min/day	113/1,367	21.2	1.02	(0.73–1.43)	0.910	0.96	(0.68–1.35)	0.813	0.90	(0.64–1.28)	0.561
	305/3,988	19.5		Trend	0.256		Trend	0.345		Trend	0.571

CI, confidence interval; HR, hazard ratio; METs, metabolic equivalents; MVPA, moderate-to-vigorous physical activity.

Model 1: Adjusted for baseline age and sex.

Model 2: Adjusted for variables in model 1 plus district, living situation, marital status, education, equivalent income, body mass index, hypertension, dyslipidemia, heart disease, stroke, diabetes mellitus, alcohol drinking status, smoking status, lower-back pain, and knee pain.

Model 3: For MVPA, adjusted for variables in model 2 plus sitting time. For sitting time, adjusted for variables in model 2 plus MVPA.

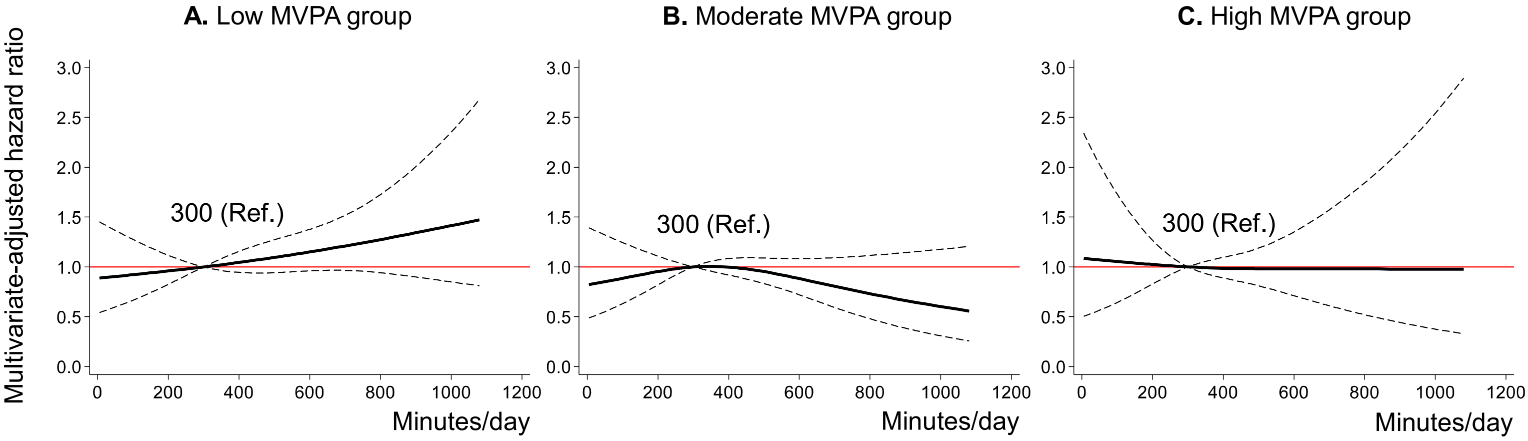

eFigure 1. Dose-response relationship of sitting time with all-cause mortality across the MVPA categories: low (**A**), moderate (**B**), and high (**C**) MVPA were <600 METs・minute/week, 600–3,000 METs・minute/week, and >3,000 METs・minute/week, respectively. All models were adjusted for baseline age, sex, district, living situation, marital status, education, equivalent income, body mass index, hypertension, dyslipidemia, heart disease, stroke, diabetes mellitus, cancer, alcohol drinking status, smoking status, lowerback pain, and knee pain. The reference values for sitting time were 300 minutes/day. The solid lines indicate the hazard ratios for all-cause mortality. The dashed lines indicate the 95% confidence intervals. METs, metabolic equivalents; MVPA, moderate-to-vigorous physical activity.

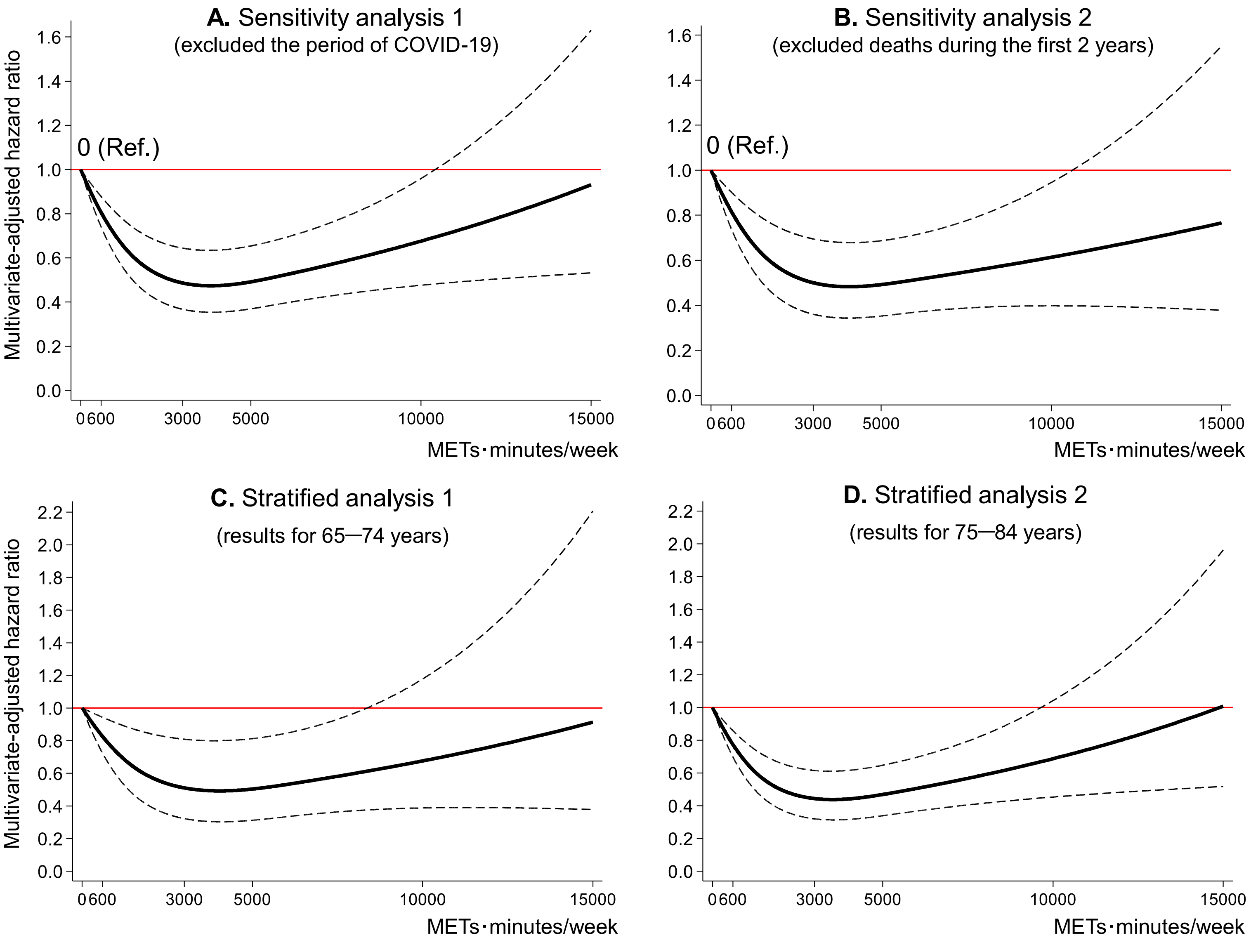

eFigure 2. Sensitivity and stratified analyses of dose-response relationships of MVPA with all-cause mortality. **eFigure 2** shows the results of the sensitivity analyses after (**A**) excluding the period of the COVID-19 pandemic (n=8,069) and (**B**) excluding deaths during the first 2 years of follow-up (n=7,901), as well as the stratified analyses by two age groups of (**C**) 65–74 years (n=4,081) and (**D**) 75–84 years (n=3,988). All models were adjusted for the baseline age, sex, district, living situation, marital status, education, equivalent income, body mass index, hypertension, dyslipidemia, heart disease, stroke, diabetes mellitus, cancer, alcohol drinking status, smoking status, lower-back pain, knee pain, and sitting time. The reference value for each model was 0 METs·minutes/week. The solid lines indicate the hazard ratios for mortality. The dashed lines indicate the 95% confidence intervals. COVID-19, novel coronavirus disease 2019; METs, metabolic equivalents; MVPA, moderate-to-vigorous physical activity.

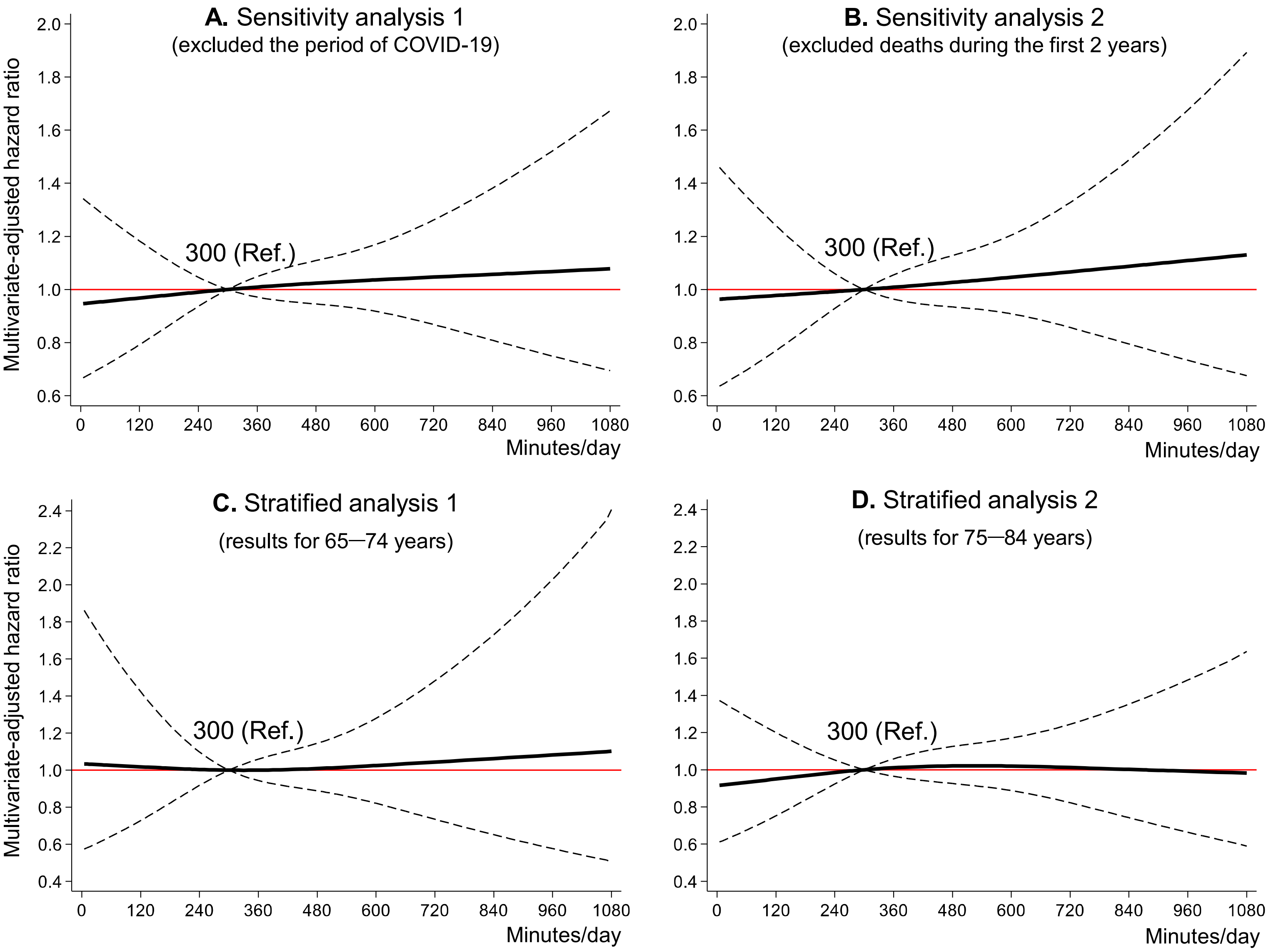

eFigure 3. Sensitivity and stratified analyses of dose-response relationships of sitting time with all-cause mortality. **eFigure 3** shows the results of the sensitivity analyses after (**A**) excluding the period of the COVID-19 pandemic (n=8,069) and (**B**) excluding deaths during the first 2 years of follow-up (n=7,901), as well as the stratified analyses of two age groups of (**C**) 65–74 years (n=4,081) and (**D**) 75–84 years (n=3,988). All models were adjusted for the baseline age, sex, district, living situation, marital status, education, equivalent income, body mass index, hypertension, dyslipidemia, heart disease, stroke, diabetes mellitus, cancer, alcohol drinking status, smoking status, lower-back pain, knee pain, and MVPA. The reference value for each model was 300 minutes/day. The solid lines indicate the hazard ratios for mortality. The dashed lines indicate the 95% confidence intervals. COVID-19, novel coronavirus disease 2019; MVPA, moderate-to-vigorous physical activity.
